# Exploring the Genetic Basis of Variation in Gene Predictions with a Synthetic Association Study

**DOI:** 10.1371/journal.pone.0011645

**Published:** 2010-07-29

**Authors:** Tera C. Levin, Andrew M. Glazer, Lior Pachter, Rachel B. Brem, Michael B. Eisen

**Affiliations:** 1 Department of Molecular and Cell Biology, University of California, Berkeley, California, United States of America; 2 Department of Mathematics, University of California, Berkeley, California, United States of America; 3 Howard Hughes Medical Institute, University of California, Berkeley, California, United States of America; University of California Riverside, United States of America

## Abstract

Identifying DNA polymorphisms that affect molecular processes like transcription, splicing, or translation typically requires genotyping and experimentally characterizing tissue from large numbers of individuals, which remains expensive and time consuming. Here we introduce an alternative strategy: a “synthetic association study” in which we computationally predict molecular phenotypes on artificial genomes containing randomly sampled combinations of polymorphic alleles, and perform a classical association study to identify genotypes underlying variation in these computationally predicted annotations. We applied this method to characterize the effects on gene structure of 32,792 single-nucleotide polymorphisms between two strains of the antibiotic producing fungus *Penicilium chrysogenum*. Although these SNPs represent only 0.1 percent of the nucleotides in the genome, they collectively altered 1.8 percent of predicted gene models between these strains. To determine which SNPs or combinations of SNPs were responsible for this variation, we predicted protein-coding genes in 500 intermediate genomes, each identical except for randomly chosen alleles at each SNP position. Of 30,468 gene models in the genome, 557 varied across these 500 genomes. 226 of these polymorphic gene models (40%) were perfectly correlated with individual SNPs, all of which were within or immediately proximal to the affected gene. The genetic architectures of the other 321 were more complex, with several examples of SNP epistasis that would have been difficult to predict *a priori*. We expect that many of the SNPs that affect computational gene structure reflect a biologically unrealistic sensitivity of the gene prediction algorithm to sequence changes, and we propose that genome annotation algorithms could be improved by minimizing their sensitivity to natural polymorphisms. However, many of the SNPs we identified are likely to affect transcript structure *in vivo*, and the synthetic association study approach can be easily generalized to any computed genome annotation to uncover relationships between genotype and important molecular phenotypes.

## Introduction

As the cost of DNA sequencing plummets it is increasingly possible to sequence multiple genomes from a single species to identify naturally occurring polymorphisms. However, characterizing the phenotypic consequences of specific variants remains a significant challenge.

A crucial first step in this process is to understand how polymorphisms alter molecular phenotypes, such as protein-coding gene location, structure, and expression pattern. While the effects of some sequence changes, such as the gain or loss of a stop codon, are predictable, the overall relationship between sequence changes and gene structure remains unresolved.

Genome-wide association studies (GWAS), which have been highly effective at identifying polymorphisms that have an effect on disease and other organism level phenotypes [1], [2], [3], are increasingly being used to link polymorphisms to molecular phenotypes [4], [5]. But the expense and time required to generate data for such molecular GWAS studies limit their widespread use.

Here we present a strategy that associates naturally occurring polymorphisms with variation in inferred molecular phenotypes – in this case computationally predicted protein-coding genes. This approach has several clear advantages. Most significantly, it does not require experimental analysis of hundreds or thousands of samples. Furthermore, the use of a computed phenotype allows us to circumvent the need for a large population of genotyped or sequenced individuals. Instead, we computationally phenotype a large population of “intermediate” genomes, each containing a random sample of alleles from a set of polymorphisms identified from a natural population. This design allows us to get strong statistical power to detect associations by arbitrarily increasing the number of intermediate genomes analyzed.

Because collections of predicted transcripts only partially capture the complexity of real transcriptomes, we expect many of the variable gene models and associated polymorphisms not to be relevant *in vivo*. However, gene prediction programs and other tools for computational annotation are becoming increasingly accurate, and thus our synthetic association study is likely to identify many polymorphisms that are good candidates for experimental investigation. We also believe that probing the sensitivity of gene prediction programs to naturally occurring polymorphism will help improve these and other computational annotation algorithms that are becoming increasingly important in biology.

## Results

### Identifying gene model changes

*Penicillium chrysogenum*, the fungus originally identified by Alexander Fleming as the source of penicillin, remains an important commercial source of this antibiotic. During the past 60 years, industrial strains of *P. chrysogenum* have undergone many rounds of mutagenesis and selection in order to improve drug production [6]. Two genomes of *P. chrysogenum* have been sequenced: one from an industrial strain, here termed “vdB” [7], and one from a natural isolate of the fungus that we sequenced as part of a graduate class on genome sequencing at UC Berkeley, here termed “UCB”.

While assemblies were available for both strains, we wanted to compare gene predictions between the two genomes without anomalies arising from chromosomal rearrangements or assembly errors. We therefore identified 32,792 well-supported SNPs between the two strains by aligning reads from UCB against the vdB assembly. We used these SNPs to generate a new genome sequence, vdB*, which was identical to vdB except that all of the SNP positions were mutated to the UCB allele.

**Figure 1:**
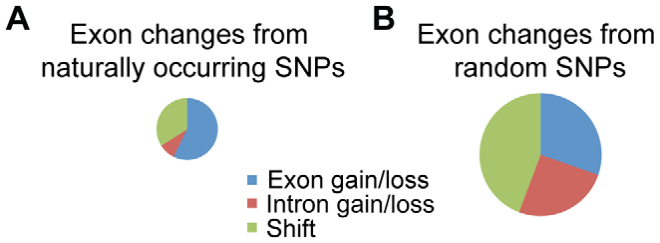
Naturally occurring SNPs cause fewer gene model differences than randomly placed SNPs. A) Gene model differences between vdB and vdB*, where all base pair changes were made at naturally occurring SNP positions between UCB and vdB. B) Average gene model differences between vdB and 100 genomes in which SNPs were randomly relocated. Area in circle is proportional to the number of changes observed.

To determine the effects of these SNPs on gene predictions, we ran the gene prediction program SNAP [8] with identical parameters on the vdB and vdB* genomes. Even though vdB and vdB* differed at only 0.1% of bases, 688 of the exon predictions (1.8%) were variable between the two genomes. These included 234 “shifts”, in which the 5′ and/or 3′ boundaries of exons were altered; 59 “intron gain/losses”, in which two exons in one genome were predicted to be a single, large exon in the other genome; and 395 “exon gain/losses”, in which an exon predicted in one genome was absent in the other genome (Figure 1A).

Variable exons had lower SNAP scores (23±33) than non-variable exons (44±60) (p = 10^−38^ by two-tailed t-test). This suggests that exons that are less confidently determined by SNAP may be more prone to vary in response to SNPs. However, given the high degree of overlap between the SNAP score distributions of variable and non-variable exons, it is not possible to predict *a priori* whether an individual SNAP gene model is sensitive to sequence polymorphisms.

### Random sequence differences affect gene models more than naturally occurring polymorphisms

We were initially surprised to observe that such a high percentage of exons (1.8%) vary between vdB and vdB*. To determine how this compared to what might be expected by chance, we generated 100 versions of the vdB genome with randomly positioned mutations. The same number and types of base pair changes were made in these randomized genomes as between the vdB and vdB* genomes.

There were over twice as many gene model changes between vdB and the randomized genomes (mean = 2651, std. dev. = 93) as between vdB and vdB* (1032) (Figure 1B). Thus, although there are many exon changes between vdB and vdB*, there are many fewer changes than would be expected from random mutations, suggesting that purifying selection has acted to remove many mutations that would alter gene models. This result also suggests that SNAP predictions are sensitive not just to the particular polymorphisms between vdB and vdB*, but to SNPs in general.

Between the vdB and vdB* genomes, we were unable to distinguish between an exon gain and an exon loss (or intron gain versus intron loss) without an outgroup to determine the ancestral SNP allele. However, for the defined mutations of the randomized genomes, we found that there were more exon losses than exon gains (259+/−19 exon losses versus 154+/−16 exon gains) and more intron gains than intron losses (309+/−16 intron gains versus 37+/−6 intron losses). Therefore, at least with random mutations, it is more common to lose exons and gain introns.

### A synthetic association study

We next wanted to investigate the mechanism by which SNPs were affecting gene predictions. To do this, we first needed to identify which SNPs were affecting which SNAP predictions. This proved difficult because: 1) it was unknown if a gene prediction was affected by a single SNP or multiple SNPs; 2) there were often many SNPs close to and within the altered gene model; and 3) many of the altered gene predictions did not have SNPs in obviously functional sites (e.g. a stop codon).

To overcome these difficulties, we set out to use an “*in silico* genetics” approach, in which we computationally mutated genomes and examined the resulting SNAP phenotypes. One possible approach would be to generate all 32,792 possible single-mutant genomes, each identical to vdB except at a single SNP, to see if one of the single mutants phenocopied vdB* at a specific locus. However, running SNAP on this many genomes would take an inordinate amount of computational time. Moreover, in order to find cases where 2, 3, or more SNPs together influenced a single gene, one would additionally need to generate double, triple, etc. mutant genomes.

**Figure 2:**
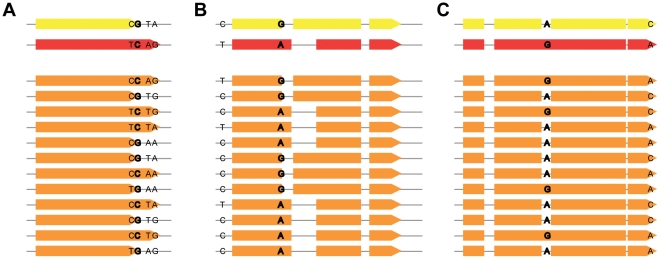
Using intermediate genomes to identify correlations between SNPs and gene model events. Comparison of exon models from vdB (red), vdB* (yellow), and intermediate (orange) genomes. All SNPs in the region are shown, with the SNP that perfectly correlates with the event in bold. A) A “shift” event. A SNP in a *TGA* stop codon in vdB* is associated with a shift in exon length on contig 16: 3577500–3576100. B) Two “shift” events. A novel splice donor in vdB* affects the length of its exon and a neighboring exon on contig 20: 2945150–2945750. C) An “intron gain/loss” event. An intronic SNP affects the presence/absence of an intron on contig 12: 69800–70950.

As a more effective and tractable alternative, we devised a strategy that we refer to as a synthetic association study. A typical association study examines the relationship between genotype and phenotype within a population of individuals whose genomes have been shuffled by an extended period of meiotic recombination. In our synthetic association study, we computationally shuffled the SNP genotypes of vdB and vdB*, simulating 500 genomes that were intermediate in genotype to vdB and vdB*. Specifically, at every SNP position, each intermediate genome had a 50% chance of incorporating either the vdB or UCB allele. For each of these 500 intermediate genomes, we ran SNAP to generate the gene prediction phenotype and then looked for associations between specific SNP genotypes and gene model phenotypes (Figure 2).

### Gene model phenotypes with perfectly associated SNPs

**Figure 3:**
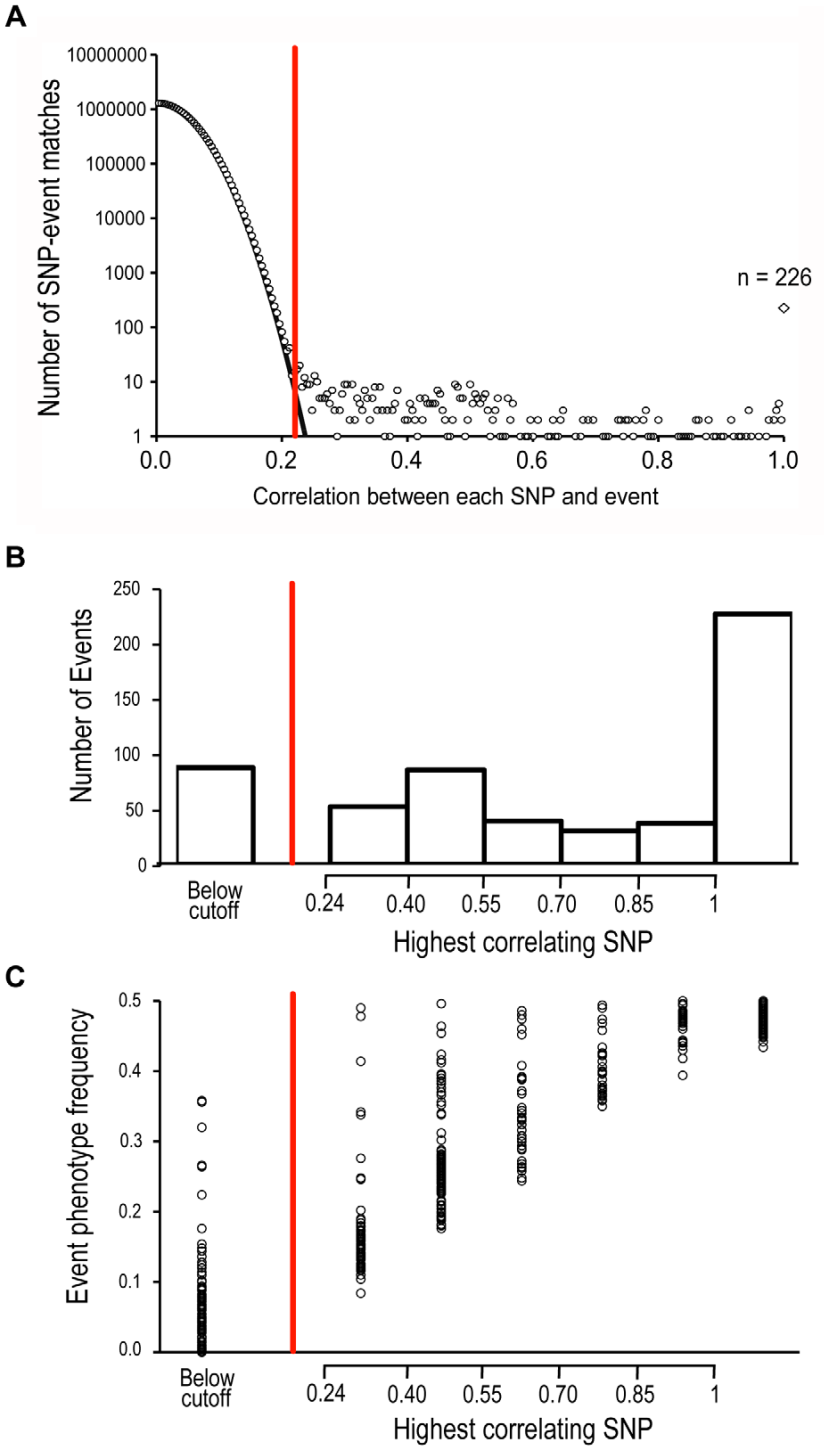
Most events are significantly correlated with one or more SNPs. A) Histogram of the correlation between every pair-wise event-SNP combination. A correlation of 1.0 (diamond marker) indicates perfect agreement between the SNP genotype and event phenotype in all intermediate genomes. The black line shows a theoretical binomial distribution of correlations between independent events and SNPs. B) Histogram showing the largest SNP-event correlation for each event. C) The event phenotype frequency, the fraction of intermediate genomes containing the less common phenotype of each event, binned as in B. For all graphs, the significance cutoff is shown by the red line.

As multiple exons often changed in a coordinated manner, we grouped 2 or more polymorphic exons into an “event” when they co-occurred 100% of the time in the intermediate genomes. For example, in Figure 2B four variable exons are grouped into an event because they occur in a completely correlated pattern in the intermediate genomes. We found 557 total events. We then looked for associations between events and SNP alleles. We observed many cases in which a single SNP allele co-occurred with an event phenotype in all 500 intermediate genomes. We interpreted this association to mean that this single SNP was responsible for the altered gene prediction phenotype. We performed a genome-wide search for such cases by looking for correlations between the occurrence of each event and each SNP allele in the 500 intermediate genomes. We found 226 events that completely correlated with the presence of a particular polymorphic allele (Figure 3). Each of these event-SNP associations was highly significant (false discovery rate = 10^−103^). Encouragingly, all of the perfectly correlating SNPs were local to their associated events. Most SNPs were located within the boundaries of the event (89%), while the remaining SNPs were external to the event but nearby (median of 11 bp away). As hidden markov model (HMM) gene predictions are expected to be sensitive primarily to local perturbations, these results lend confidence to the synthetic association study method.

**Figure 4:**
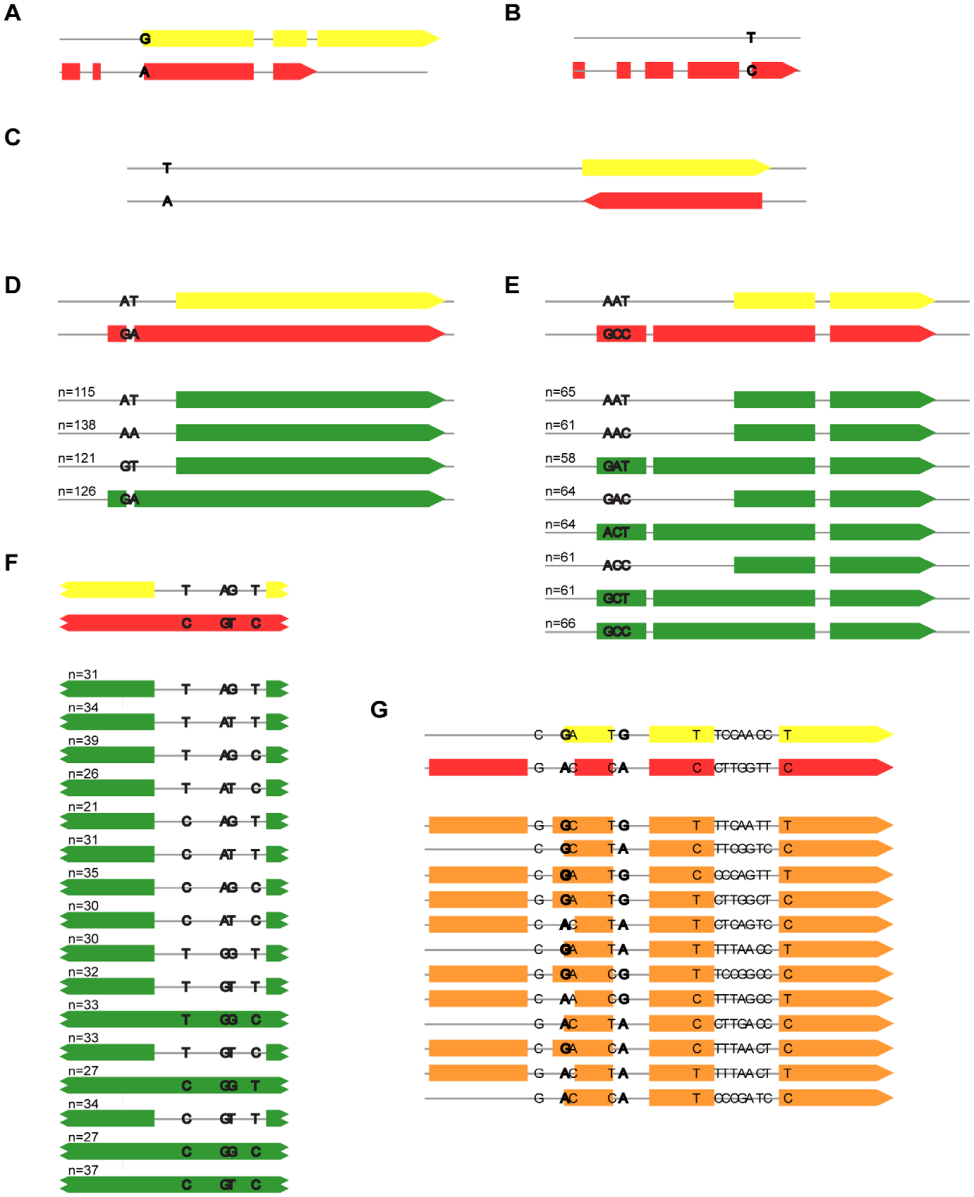
A variety of SNP genetic architectures affect exon model phenotypes. A–C) Diverse gene model phenotypes associated with a single SNP. A) A SNP is part of a start codon in vdB* and a splice acceptor in vdB on contig 21: 133200–133500 B). An entire five-exon gene is predicted in vdB but not vdB* on contig 24: 56700–57500. C) A rare example in which an intergenic SNP causes a gene to flip strands on contig 22: 2627100–2627839. D–F) Examples of events that are completely explained by multiple SNPs. All possible SNP combinations and associated phenotypes are shown in green. D) One SNP is part of a splice donor and a second SNP is part of a splice acceptor. Both must be present for the additional exon to be predicted. Contig 19: 82900–82200. E) Three exonic SNPs affect the presence of the first exon and the length of the middle exon. Contig 24: 503000–504500. F) Four intronic SNPs explain the presence/absence of an intron. Contig 22: 2204700–2204850. G) An example of an event that is not completely explained. The two SNPs in bold are significantly correlated with the event, but examining those SNPs alone does not fully explain the phenotype. We also observe a gene model that contains a third phenotype with a longer second exon, unobserved in either vdB or vdB*. Contig 20: 3227900–3228500.

To better understand how these perfectly correlating SNPs affected gene models, we examined the positions of the SNPs relative to their associated events. The SNPs were often located in “influential sites” in a gene, defined here as the start codons, stop codons, splice donor, and splice acceptor sequences (Table 1). 92 out of 226 (41%) perfectly associated SNPs were in influential sites in at least one of the two genomes. In the remaining cases, the perfectly associated SNP was not in an influential site in either genome. These SNPs were found in exons (n = 73, e.g. Figure 4B) or introns (n = 59) of vdB or vdB*, and occasionally they were located in intergenic regions in both genomes (n = 2, e.g. Figure 4C). The mechanisms by which SNPs in non-influential sites affect gene models remain unclear.

**Table 1:** Locations of SNPs that perfectly associate with gene models.

	Intron gain/loss (n = 28)	Exon gain/loss (n = 122)	Shift (n = 116)	Total (n = 226)
Start codon	0	11	14	19
Stop codon	18	17	22	54
Splice donor	1	5	9	14
Splice acceptor	1	2	2	5
Other	8	87	69	134

Note that as each event can belong to one or more category (Intron gain/loss, Exon gain/loss, and/or Shift), the Total is not necessarily equal to the sum of the three categories.

### Gene model phenotypes affected by modifier SNPs

In addition to the 226 perfect correlations between events and SNPs, there were also many cases of SNPs and events that were highly, but not perfectly, correlated. We interpret these correlations to mean that there are modifier SNPs that affect but are not completely responsible for the phenotype of that event.

To determine which of these SNP-event correlations were significant, we compared the distribution of correlations to a theoretical model in which all SNPs and events were unassociated. Unassociated SNPs and events are predicted to have a distribution of observed correlations that peaks at zero and extends as a binomial distribution. The distribution of event-SNP correlations in the association study fit this binomial curve for correlations between 0 and 0.2, but diverged for higher correlation values (Figure 3A). After we set a false discovery rate cutoff of 5%, all correlation values above 0.244 were significant. This analysis identified 438 significant, non-perfect SNP-event correlations in addition to the 226 perfect correlations. All of these SNPs were also near their associated event. Overall, we were able to detect an average of 1.2 significant SNPs per event.

We next asked if the exon model variation was due to a small number of genotypic changes (some with a large effect) or many changes (each with a small effect). 226/557 (41%) events were completely controlled by a single SNP (Figure 3B). 243 events (44%) were associated with multiple SNPs of intermediate effect, at least one of which had a large enough correlation value to be significantly detected in this study. In contrast, 88 events (15%) had no significant SNP correlations, suggesting that these events were influenced by many SNPs, each with a small effect below the significance cutoff. Thus, the overall phenotypic difference between vdB and vdB* was due to a combination of large, intermediate, and small-effect SNPs.

### Complex traits and SNP epistasis

Of the events associated with multiple SNPs, we observed several cases in which an event could be “completely explained” by 2, 3, or 4 SNPs, meaning that the genotype at only the 2, 3, or 4 SNPs was relevant for predicting the gene model phenotype (Figure 4D–F). We performed a systematic search for such events and identified events that could be completely explained by 2 SNPs (n = 37), 3 SNPs (n = 17), and 4 SNPs (n = 1). Some of these are clear examples of complex SNP epistasis. For example, in Figure 4F, an intron is always present when the second bolded SNP is A, but when the second SNP is G, an intron may be present or absent depending on the other SNP alleles. This variety of genetic architectures highlights the complexity and unpredictability of SNAP.

Overall, we found all of the SNPs that control an event for only 50% (n = 280) of the events (n = 226 one SNP events, n = 37 two SNP events, n = 16 three SNP events, n = 1 four SNP event). Thus, it is common for events to have one (or more) small, modifier SNPs that are below the significance cutoff of 0.244, where we are unable to detect them (Figure 4G).

Traditional association studies have trouble identifying causative alleles when the phenotype of interest is examined in a small number of individuals, or when the alleles that influence the phenotype are of small effect [9]. Analogously, we observed that for many of the incompletely explained events, one event phenotype was much more common in the intermediate genomes, and the other version appeared only rarely (Figure 3C). Completely explained events had an average phenotype frequency of 48.2%, appearing in 241 out of 500 intermediate genomes. In contrast, the events that had no significantly correlated SNPs had an average phenotype frequency of 7.6%, corresponding to only 36 out of 500 intermediate genomes, which was too few to find significant associations. Thus, it appears that our synthetic association study falls victim to some of the same weaknesses as traditional association studies. However, these difficulties can be overcome by increasing the number of individuals analyzed. While traditional association studies are often limited by the time and cost required to collect individuals from the wild and to assess their genotypes and phenotypes, the number of individuals in synthetic association studies is limited only by computational power.

## Discussion

### The utility of synthetic association studies

Computational methods play an important role in genome annotation. To the extent that these methods accurately capture biological reality, they provide a powerful means to understand the molecular consequences of sequence variation within and between species. However, as these methods have grown more accurate, they have also grown more complex, to the point where it is no longer possible to predict their behavior.

To study the unpredictable behaviors of these complex models – and hopefully the biology they represent – we have co-opted techniques developed to study complex organisms. Association studies analyze a population of individuals to understand the ways in which genes influence phenotypes. Here, we present the concept of a synthetic association study for analyzing the behaviors of complex genomic programs. Since the phenotype we examined was strictly computational, we could easily phenotype an arbitrarily large synthetic population of intermediate genomes to understand how sequence polymorphisms produce variable gene predictions. (Strictly speaking, our approach is analogous to an association study with zero linkage between neighboring SNPs, allowing us to precisely identify the specific SNP or SNPs that affect a gene model.)

The synthetic association study is not limited to gene prediction programs, and could be extended to understand any complex biological phenomenon that is modeled by a complex program. We discovered at least one SNP that was significantly associated with 85% of variable gene predictions. Additionally, we identified many cases in which 2, 3, 4, or more SNPs were responsible for the gene model phenotype, demonstrating that SNPs can affect gene predictions in a complex and combinatorial manner.

### How biologically accurate are SNAP responses to SNPs?

Of the 226 SNPs that were perfectly correlated with a variable gene model, 92 (41%) were located in obviously influential sites within a gene (e.g. start and stop codons, splice sites). To the extent that the affected genes exist, which we believe most do, these SNPs are likely to affect their structure *in vivo*.

The remaining 134 SNPs were found in the middle of exons, introns, or intergenic regions. While sequence motifs inside exons and introns are known to influence gene boundaries through the action of splicing regulators that bind these sites, these effects were not explicitly modeled in the HMM used in SNAP [8], [10]. However, SNAP does consider the composition of each sequence element in computing the likelihood of particular gene models, and sequence changes within these elements may affect the likelihood of alternative gene models or states (e.g. no gene) enough to alter SNAP's predictions. Since SNAP is trained to recognize real genes, some of this behavior may be real. But some behaviors of SNAP that we observed are unlikely to accurately reflect biological differences between the strains. For instance, we observed 2 cases where a single SNP caused the SNAP prediction to “flip” from one strand to the other (Figure 4C). Overall, 269 of the 557 (48.3%) polymorphic events are influenced by at least one SNP located in a non-influential site. These events (representing 1.1% of the gene predictions in the genome) are the most likely to be erroneous.

### Assessing gene predictions without validated gene sets

Any improper sensitivity of SNAP to polymorphisms should be of serious concern to those who wish to use gene predictions for subsequent experiments or genome analyses, especially given that SNAP was additionally sensitive to random polymorphisms that could result from random mutation or sequencing error. We therefore suggest that inappropriate sensitivity to polymorphism should be a key criterion of gene model program assessment. Currently, analyses of the accuracy of gene prediction programs rely heavily on comparisons between gene predictions and experimentally validated gene sets in model organisms [8], [11]–[16]. These approaches are not always available to evaluate gene predictions in non-model organisms, as validated gene sets are often non-existent. In these situations, gene prediction programs are evaluated by comparing the outputs of different programs to each other [17], but this process is at risk of missing shared errors. The synthetic association study can fill this gap of gene prediction assessment in non-model genomes by identifying potentially sensitive, problematic gene predictions. The synthetic association study could even be used in the process of program optimization, with the goal of minimizing the number of gene predictions that are sensitive to natural variation. Although this is admittedly a less reliable procedure than using validated gene sets, the rate of sequencing new genomes is far outpacing the rate of developing new validated gene sets. We will need assessment methods, such as the synthetic association study, that can pinpoint specific defects in gene models (or other bioinformatic outputs) in the absence of experimental validation.

## Methods

### SNP identification

SNPs were identified using MosaikAligner [18] to map the UCB raw sequencing reads to the vdB genome. SNP calls were made in regions of 5× or more read coverage when the UCB reads contained a different single base from the aligned vdB genome.

### SNAP parameter training on the *P. chrysogenum* genome

Beginning with the *C. elegans* SNAP parameters, we ran SNAP on the vdB FASTA file to create a .zff training data file. We took the output of this run to use as the input parameters for another SNAP training run on the vdB FASTA file. This cycle was repeated for 25 training rounds to optimize the SNAP parameters for *P. chrysogenum*.

### Generating the vdB* genome

Beginning with the vdB FASTA file, we altered every SNP position to contain the UCB instead of the vdB allele. Thus, the vdB* genome has the vdB genome as a backbone, and is different at only 32,792 bases (0.1% of the genome).

### Creating intermediate genomes and gene models

Intermediate genomes also have the vdB genome as a backbone. At every SNP position, we randomly incorporated (with a 50% chance) either the UCB allele or the vdB allele. Thus, SNP alleles are independent and there is no linkage.

Subsequently, SNAP was run on all intermediate genomes using the previously described parameters (see ‘SNAP parameter training’). We then determined the presence or absence of every exon model that was predicted in any of the SNAP runs. This allowed for the possibility of observing exons in intermediate genomes that were not predicted in either the vdB or vdB* genomes. Exon models were defined by their contig, type (e.g. initial exon, terminal exon), start position, stop position, and strand.

### Defining and characterizing events

Exon changes that were completely correlated or completely anti-correlated in their patterns of presence and absence in the vdB, vdB*, and the 500 intermediate genomes were grouped together into an “event.”

As we were interested in understanding the variation between vdB and vdB*, we only considered events which were polymorphic between these two genomes. 99.8% of these events contained grouped exons that were all on the same contig (557/558); the single event that grouped exons from multiple contigs was removed from the analysis.

In cases where multiple exons in the same genome were grouped into a single event, 90% of the grouped exons were in the same gene, and 10% were in neighboring genes, demonstrating that grouped exons tended to be local.

### Searching for and characterizing associations between SNPs and event phenotypes

We searched for correlations between the patterns of event presence/absence and the occurrence patterns of every SNP. Some SNPs-event pairs (n = 226) perfectly co-occured, while many were more weakly correlated, as seen in Figure 3. Non-correlated SNP-event pairs form a distribution that mirrors a theoretical calculation generated using a binomial distribution.

We converted the number of times a SNP allele co-occurred with an event into a “correlation”, defined as:
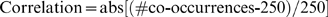



This formula is a linear conversion that converts a SNP-event agreement frequency of 500 into a correlation of 1, and a SNP-event agreement frequency of 250 into a correlation of 0.

### Categorizing events

Using the start and stop positions of each exon, we determined the overlap between pairs of exons in vdB and vdB*. Exon gain/loss changes were defined as single exons that overlapped no exons in the other genome. Intron gain/loss changes were defined as single exons that overlapped 2 or more exons in the other genome. Shift exon changes were defined as a single pair of overlapping exons that had different start and/or stop sites. Finally, “flip” exon changes were defined as any case where exons in the same event were positioned on opposite strands. More extreme changes were considered to be hierarchically more important than other changes; for example flip events were not further analyzed for the other categories of change, and an exon change that could be analyzed as both a join/split event and a shift was considered to be a join/split change, because this was the more extreme change. Individual events could consist of multiple changes, for example, a single event could have both a “shift” change and an “exon gain/loss” change involving different exons.

### Generating a genome with randomized SNP locations

We began by assessing the number of SNP polymorphisms of each type (e.g. number of A→T transitions) and by counting the number of As, Ts, Cs, and Gs in the vdB genome that had at least 5× coverage by the UCB reads. We then mutated the vdB genome such that at every base there was a small probability of mutation, based on how many mutations we had already made and how much of the genome remained un-mutated. For example, at every A in the vdB genome, the probability of mutating to a G was:
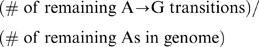
Similar probabilities were calculated for all other possible mutations in the genome, based on the number of base changes observed in the SNP calls. This procedure allowed us to generate a genome based on the vdB genome that was altered to a similar extent as vdB* strain, but with the mutation positions randomized. We created 100 of these genomes and compared the SNAP predictions from these genomes with the gene models for vdB.

To categorize gene model changes in a randomized genome, we identified all gene model predictions that were different between the vdB genome and the randomized genome. We next determined which exons in one genome overlapped which other exons in the second genome. If an exon in one genome overlapped no exons in the other genome, we classified this situation as an “exon gain/loss.” If an exon from each genome overlapped each other and only each other, we classified this situation as a “shifted exon.” Finally, if two exons in one genome overlapped a single exon in the other genome, we classified this situation as an “intron gain/loss.” This analysis was a similar classification system as that used for events, but used a distinct method. This method did not rely on any grouping of exons into events.

### Defining SNP positions relative to exons

If a SNP was within the first or last 3 base pairs of a gene, it was classified as being in the start or stop sites, respectively. If a SNP was located in an intron but within 5 base pairs of the beginning or the end of the intron, is was classified as being in a splice donor or splice acceptor site, respectively. Otherwise, a SNP was classified as intronic, exonic, or intergenic, depending on its location.

A SNP was defined relative to a pair of genomes based on a hierarchy of functional locations. For example, a SNP that was located in a start codon in vdB genome and in an intron in vdB* was classified as a start codon SNP, because its position in the start codon was considered more likely to reflect a functional role. The hierarchy of SNP positions was defined as follows: start, stop>splice donor, splice acceptor>exon>intron>intergenic.

### Searching for events completely explained by multiple SNPs

An event was considered “completely explained” if each combination of SNP alleles was always associated with a single event phenotype. For example, in Figure 4D, every time the SNPs in bold were AT, AA, or GT, the vdB* gene model phenotype resulted. Every time there was GA, the vdB gene model was predicted. Incompletely explained events, as in Figure 4G, had a single combination of SNP alleles (e.g. GG) co-occurring with multiple different gene model phenotypes. We performed a systematic search for any events that could be explained by 2, 3, 4, or 5 SNPs by looking for combinations of significant SNPs that were always associated with a single phenotype. Only significantly correlated SNPs (correlation>0.244) were tested.

### Calculating the event phenotype frequency

The event phenotype frequency was defined as the percentage of the intermediate genomes that contained the less common version of the event. Thus, events that had each version occur 250/500 times had a frequency of 0.50 and events that had one version occur either 0 or 500 times had a frequency of 0.00.
